# Telocytes contribute to aging-related modifications in the prostate

**DOI:** 10.1038/s41598-020-78532-7

**Published:** 2020-12-07

**Authors:** Bruno Domingos Azevedo Sanches, Guilherme Henrique Tamarindo, Juliana dos Santos Maldarine, Alana Della Torre da Silva, Vitória Alário dos Santos, Maria Letícia Duarte Lima, Paula Rahal, Rejane Maira Góes, Sebastião Roberto Taboga, Sérgio Luis Felisbino, Hernandes F. Carvalho

**Affiliations:** 1 Department of Structural and Functional Biology, Institute of Biology, State University of Campinas (UNICAMP), Bertrand Russel Av., Campinas, São Paulo Brazil; 2 Laboratory of Microscopy and Microanalysis, Department of Biology, São Paulo State University (UNESP), Cristóvão Colombo St., 2265, São José Do Rio Preto, São Paulo Brazil; 3 Laboratory of Genome Studies, Department of Biology, São Paulo State University (UNESP), Cristóvão Colombo St., 2265, São José Do Rio Preto, São Paulo Brazil; 4 Laboratory of Extracellular Matrix, Institute of Biosciences, São Paulo State University – UNESP, Prof. Dr. Antônio Celso Wagner Zanin St., 250, Rubião Júnior District, Botucatu, São Paulo 18618-689 Brazil

**Keywords:** Urology, Prostate

## Abstract

Telocytes are interstitial cells present in the stroma of several organs, including the prostate. There is evidence that these cells are present during prostate alveologenesis, in which these cells play a relevant role, but there is no information about the presence of and possible changes in telocytes during prostate aging. Throughout aging, the prostate undergoes several spontaneous changes in the stroma that are pro-pathogenic. Our study used histochemistry, 3D reconstructions, ultrastructure and immunofluorescence to compare the adult prostate with the senile prostate of the Mongolian gerbil, in order to investigate possible changes in telocytes with senescence and a possible role for these cells in the age-associated alterations. It was found that the layers of perialveolar smooth muscle become thinner as the prostatic alveoli become more dilated during aging, and that telocytes form a network that involves smooth muscle cells, which could possibly indicate a role for telocytes in maintaining the integrity of perialveolar smooth muscles. On the other hand, with senescence, VEGF+ telocytes are seen in stroma possibly contributing to angiogenesis, together with TNFR1+ telocytes, which are associated with a pro-inflammatory microenvironment in the prostate. Together, these data indicate that telocytes are important both in understanding the aging-related changes that are seen in the prostate and also in the search for new therapeutic targets for pathologies whose frequency increases with age.

## Introduction

Telocytes are interstitial cells with well-defined morphological aspects, which have long, thin cytoplasmic extensions, called telopodes. Telopodes are, in turn, divided in dilated sections, the podoms, which have mitochondria and other organelles, and the podomers, which are extended, thin segments; the alternation of these two parts gives rise to the moniliform or beaded morphology of telocytes^1,2^. These cells diverge from fibroblasts in morphology, proteome^3^ and gene expression^4,5^. Telocytes are present in the stroma of several organs and tissues, such as the heart^6,7^, the scalp^8^, mammary glands^9,10^, joints^11^, kidney^12^, duodenum^13,14^, tongue^15^, and prostate^16^, among many others, so that, given the current evidence, telocytes can be considered a typical cellular component of the stroma. Moreover, there is an increasing set of data indicating that these cells have profound interrelationships with other stromal cell types as well as with the epithelium. There is also evidence that telocytes contribute to tissue regeneration^7,17^, the maintenance of stem cell populations^18,19^, muscle contraction^20,21^, and the maintenance of the stroma in order to prevent the formation of fibrosis^22,23^, among others. These findings culminates with the positioning of telocytes as a very relevant component in both the physiology and pathophysiology of several organs.

There are findings supporting the hypothesis that telocytes participate in the physiology of the adult prostate gland, especially in supporting the contraction of perialveolar smooth muscle^16^, and that these cells are sensitive to steroidal hormones^24^. Castration promotes physiological changes in the gland, in which the telocytes undergo phenotypic transitions, becoming atrophic, as the epithelium involutes and smooth muscle cells becomes spinous and acquire the synthetic phenotype^25^. In addition, telocytes are present in this gland early in development and their association with prostate alveoli is seen in the early stages of alveologenesis^26^. These results led to the suggestion that telocytes contribute paracrinally to differentiate the perialveolar muscle layer in the prostate^24^. However, the roles played by telocytes during aging in the prostate and other organs are still unclear. Specifically, it is not known whether these cells are implicated in tissue changes that become prominent with senescence. However, there is current evidence that telocytes can increase the number of their cytoplasmic projections, telopodes, with a greater number of passages in vitro^27^, and that telocytes are implicated in the maintenance of cardiac stem cells, as these cells are found the aged heart^28^.

Prostate aging is a complex process, in which several spontaneous changes can be observed; there is much evidence that many of these changes can serve as a basis for the onset of pathogenic conditions that frequently affect the prostate with aging; such as benign prostate hyperplasia and prostate cancer^29,30^. Prostate cancer is one of the dominant causes of death in the world, which indicates that the prostate has a great propensity to develop malignant lesions^31,32^. It is not known for certain why this organ becomes so vulnerable to tissue changes. One of the possible reasons is its great sensitivity to hormonal variations, specially to changes in steroid levels, represented by an imbalance with aging of the proportion between testosterone and estradiol^33,34^. Accordingly, the testosterone:estradiol ratio declines, which mimics an environment similar to that seen in the initial development of the gland, which has high proliferative rates^33–35^ and in which the expansion of luminal cells is verified^36^. In addition, in the aging prostate, it was observed an increase of lymphocyte and macrophage infiltrates, as well as of proinflammatory factors^29,37^.

The role of stromal changes in the onset of prostate pathologies has been synthesized in the concept of reactive stroma^38–40^; even in the stage of benign lesions in the prostate, such as prostatic intraepithelial neoplasia (PINs), the stroma itself would have a series of potentially carcinogenic characteristics. In the so-called reactive stroma, the prostate stroma shows an increase in the presence of myofibroblasts, in addition to the appearance of cancer-associated fibroblasts (CAFs)^41^, and an increase in the subepithelial layer of CD34-positive fibroblast-like cells^42^, which are possible telocytes, and both these stromal cells could contribute to prostate carcinogenesis. The formation of the reactive stroma is usually related to aging, but exposure to chemical and other tumorigenic factors can aggravate or accelerate these stromal changes^43^.

Among rodents, the Mongolian gerbil is a promising species for assessing stromal changes in the prostate with aging, due to both the presence of functional prostates in females and the high incidence of spontaneous lesions^44,45^. In this scenario, the present study is an analysis of the interrelationship between telocytes and other prostate cells in the Mongolian gerbil, and considers whether these cells could contribute to the tissue changes typical of gland aging.

## Results

### Light microscopy and 3D reconstructions

**Figure 1:**
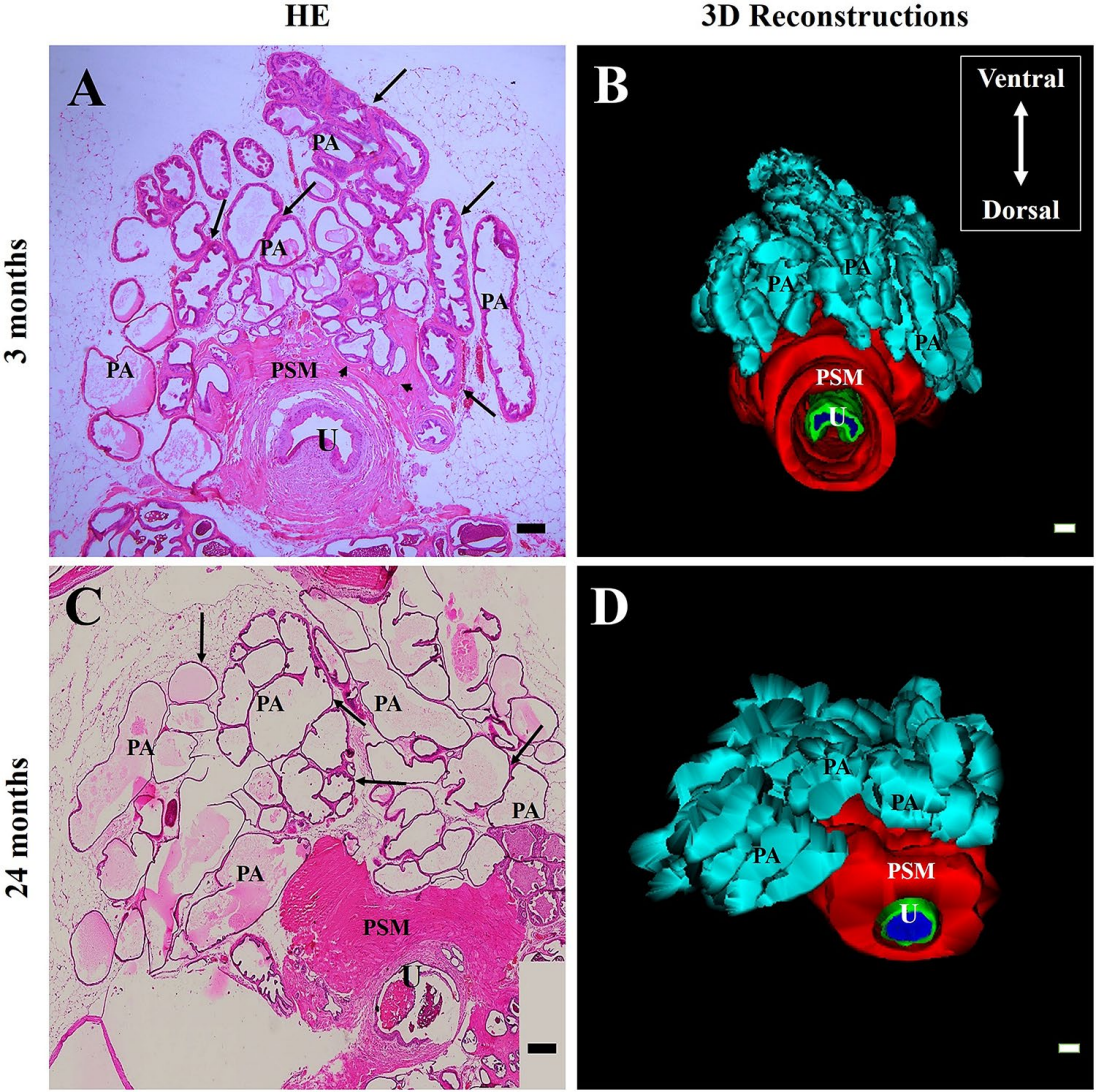
Histological sections of the adult (3 months old) and senile (24 months old) Mongolian gerbil ventral prostate stained with H&E and 3D reconstructions of these histological sections. (**A**) In the adult prostate, the prostate alveoli have thick layers of stratified smooth muscle in the perialveolar region and the periurethral smooth muscle is continuous with the perialveolar smooth muscle of some alveoli. (**B**) The epithelium of the alveoli of adult ventral prostate is often folded, which indicates intense activity of synthesis of prostate secretion. (**C**,**D**) In the elderly prostate, alveoli are dilated, usually not folded, and thin layers of perialveolar musculature are present. PA, cyan (Prostate alveoli), Arrow (Layers of smooth muscle in the perialveolar region), Arrowhead (Continuity between the periurethral and perialveolar smooth muscle layers), PSM, red (Periurethral smooth muscle), U, green (Urethra), bar (200 µm).

The light microscopy data of the adult prostate demonstrated that the prostatic alveoli have thick layers of stratified smooth muscle in the perialveolar region and that the periurethral smooth muscle is continuous with the perialveolar smooth muscle of some alveoli (Fig. 1A). In addition, the epithelium of the alveoli of the adult ventral prostate is often folded, which indicates intense prostatic secretion synthesis activity (Fig. 1B). In the elderly prostate, on the other hand, the prostatic alveoli are dilated, usually not folded, and it is possible to verify thin layers of perialveolar musculature (Fig. 1C,D).

### Ultrastructural analysis

**Figure 2:**
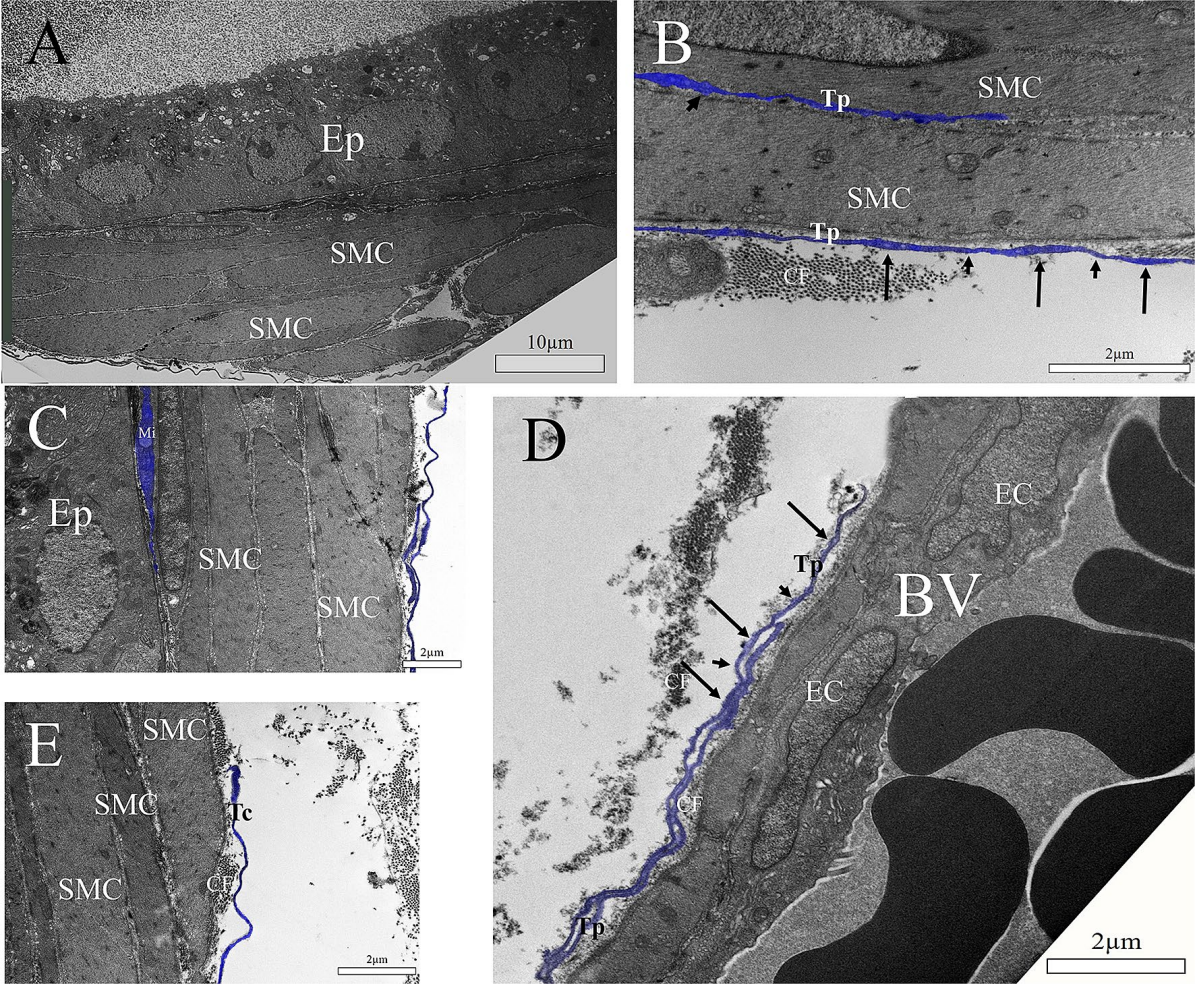
Ultrastructure of the ventral prostate of adult male gerbils (3 months old). (**A**) A stratified perialveolar smooth muscle layer can be seen; the epithelium is polarized, with vesicles going towards the apical region of the secretory cells, which is in contact with the alveolar lumen. (**B**) A telocyte can be observed in the interstitial region with close association to collagen fibrils; it is possible to observe its telopode divided into podomers and podoms. Another telopode is observed between two smooth muscle cells. (**C**) A subepithelial telocyte can be observed, which is closely associated to both epithelial and smooth muscle layers. Telopodes can be seen in the interstitium, surrounding smooth muscle cells. (**D**) Telopodes can also be seen surrounding blood vessels in close association to collagen fibrils. (**E**) Detail of an interstitial telocyte close to collagen fibrils. Ep (Prostate epithelium/epithelial cells), SMC (Smooth muscle cell), CF (Collagen fibrils), arrow (podoms), arrowhead (podomers).

In the ultrastructure of the ventral prostate of adult male gerbils (3 months of age), a layer of stratified perialveolar smooth muscle can be observed; the epithelium is polarized, with vesicles going to the apical region of the secretory cells, which is in contact with the lumen of the alveoli (Fig. 2A). A telocyte can be observed in the interstitial region in close association with collagen fibrils. Another telopode is observed between two smooth muscle cells (Fig. 2B). A subepithelial telocyte can be observed, which is closely associated to both the epithelium and the smooth muscle layer, making cellular junctions with the latter. Telopodes are present in the interstitium, surrounding smooth muscle cells (Fig. 2C), and surrounding blood vessels in close association with collagen fibrils (Fig. 2D,E).

**Figure 3:**
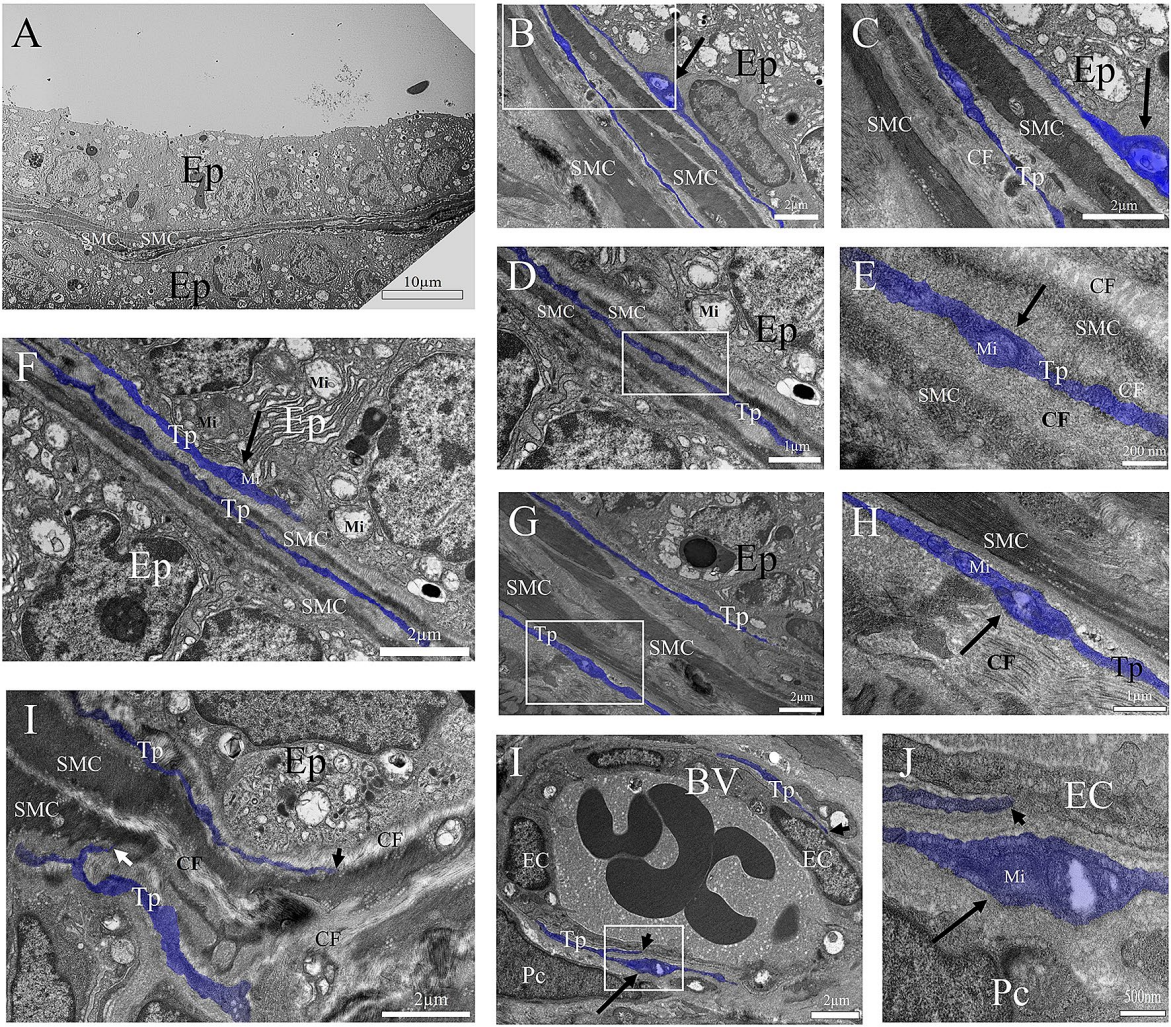
Ultrastructure of the ventral prostate of senile male gerbils (24 months of age). (**A**) A thin layer of smooth musculature surrounds the prostate epithelium. The epithelial cells have a vacuolar aspect, with dilated mitochondria, and the vesicles are distributed beyond the apical pole, at the basal pole. (**B**–**F**) Large podoms with mitochondria are seen in subepithelial telocytes; the smooth muscle layer is formed by one or two smooth muscle cells of reduced proportions and a large interspersed matrix of collagen fibrils. Telocytes divide the region between two layers of smooth musculature. (**G**–**I**) Smooth muscle cells can be seen associated with collagen fibrils. Telocytes forming a network of telopodes with can be verified in close relationship to collagen fibrils and smooth muscle cells. (**J**,**K**) Telocytes can also perform cellular junctions with endothelial cells. Ep (Prostate epithelium / epithelial cells), SMC (Smooth muscle cells), blue (telocytes), Inserts (Telocyte details), Arrow (podoms), CF (collagen fibrils), Tp (Telopode), Mi (Mitochondria), SMC (Smooth muscle cells), arrowhead (Cell junctions), EC (Endothelial cell), Pc (Pericyte).

As for the ultrastructural data of the ventral prostate of senile male gerbils (24 months of age), a reduction in the thickness of the smooth muscle layer can be observed. In addition, the epithelial cells have a vacuolated aspect, with vesicles distributed all over the cell including in the basal pole (Fig. 3A). Large podoms bearing mitochondria can be seen in subepithelial telocytes; the smooth muscle layer is formed by one or two muscle cells of reduced proportions and interspersed in a matrix of collagen fibrils; telocytes divide the region between the smooth muscle layers (Fig. 3B–F). Smooth muscle cells can be seen in association with collagen fibrils. Telocytes are observed in close relationship to collagen fibrils (Fig. 3G–I) and endothelial cells; podoms with mitochondria can be observed in these telocytes (Fig. 3J,K).

### Immunofluorescence assays

**Figure 4:**
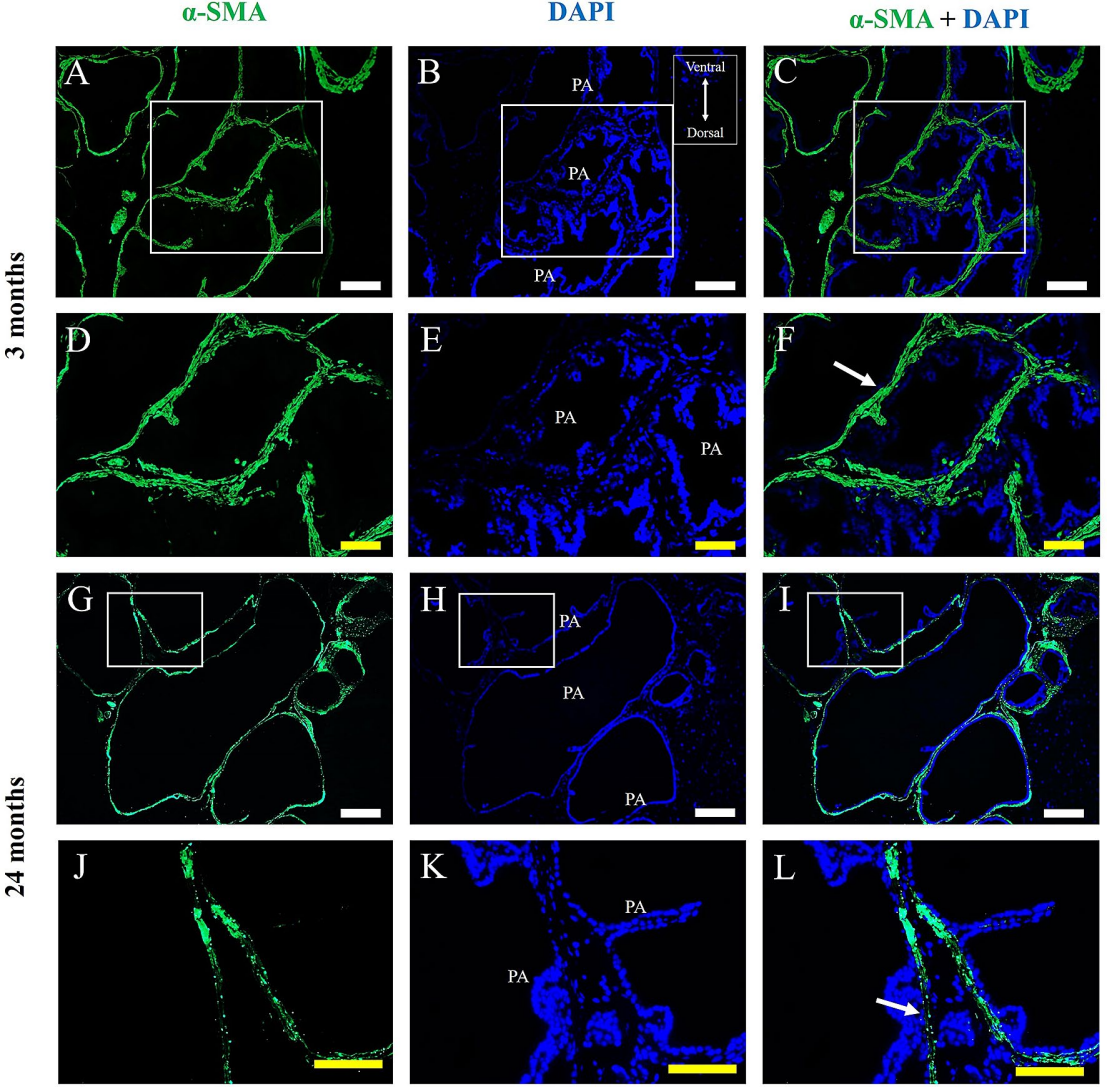
Immunofluorescence assays for α-SMA in prostate histological sections of adult (3 months old) and senile (24 months old) male Mongolian gerbils. (**A**–**F**) A thick layer of smooth muscle cells, labeled for α-SMA, surrounds the prostate alveoli. (**G**–**L**) The layer of smooth muscle cells that surrounds the alveoli becomes thinner. PA (Prostate alveoli), Arrow (Detail of smooth muscle layer), White bar (100 µm), Yellow bar (50 µm).

**Figure 5:**
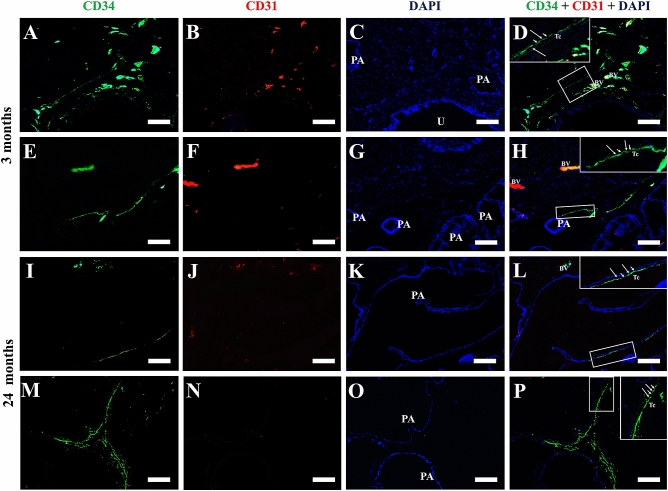
Double immunofluorescence assays for CD34 and CD31 in prostate histological sections of adult (3 months old) and senile (24 months old) male Mongolian gerbils. (**A**–**D**) The CD34 immunolabeling in adult animals (3 months of age) is found dispersed in the prostate stroma in blood vessels, in which it colocalizes with CD31. It is possible to verify a telocyte in the prostate stroma, which is CD34-positive and CD31-negative, it has a long CD34-positive telopode. (**E**–**H**) Another telocyte is observed, with a telopode reaching a prostatic alveolus. (**I**–**L**) In the prostate of senile animals (24 months of age), it is possible to observe the CD34 immunostaining typical of a telocyte in the perialveolar region. (**M**–**P**) Immunolabeling for telocytes is also observed close to the prostate alveoli in the interstitium. PA (Prostate alveoli), U (Urethra), Inserts (Detail of telocytes, with emphasis on telopodes), BV (Blood vessel), Arrow (podomers), Arrowhead (podoms), Bar (50 µm).

**Figure 6:**
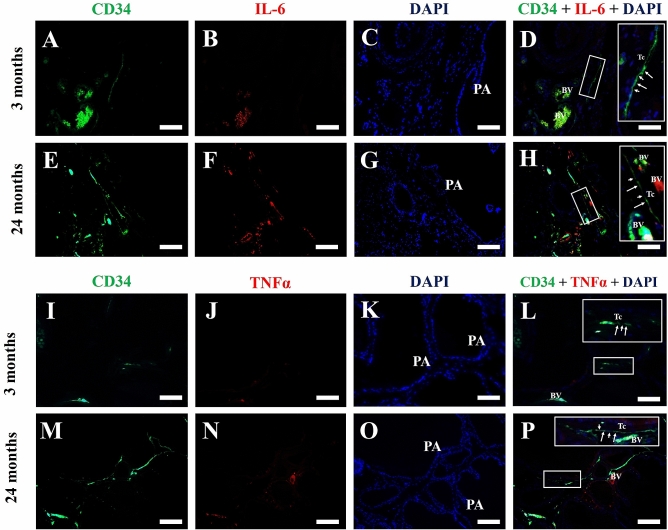
Double immunofluorescence assays for CD34 and IL-6, as well as for CD34 and TNF-α in prostate histological sections of adult (3 months old) and senile (24 months old) male Mongolian gerbils. (**A**–**D**) In the adult prostate, CD34 immunolabeling colocalizes with that of IL-6 in blood vessels, but not in telocytes. (**E**–**H**) In the prostate of senile males, CD34 immunolabeling also does not colocalize with IL-6 in telocytes only in blood vessels. (**I**–**L**) In the adult prostate, TNF-α immunolabeling colocalizes with CD34 in some blood vessels, but does not colocalize in the telocytes. (**M**–**P**) As seen in the adult prostate, the senile prostate also does not show TNF-α and CD34 colocalization in the telocytes, only in some blood vessels. PA (Prostate alveoli), Insert (Detail of a telocyte, with emphasis on telopodes), Tc (Telocyte), Bar (50 µm).

α-SMA was used in prostate histological sections of adult male Mongolian gerbil, for the detection of perialveoar smooth muscle; a thick layer of smooth muscle cells, labelled for α-SMA, can be observed surrounding the prostate alveoli (Fig. 4A–F). In the senile prostate, this layer of smooth muscle cells becomes thinner (Fig. 4G–L). In order to assess the presence of telocytes in the adult and senile prostate, double immunofluorescence assays for CD34 and CD31 were performed in histological sections of the prostate, so that the telocytes are CD34-positive and CD31-negative, which discriminates against blood vessels that are positive for both markers. In adult animals, the immunolabeling for CD34 (3 months old) is found dispersed in the prostate stroma in blood vessels, in which colocalization with CD31 is observed. It is possible to verify a telocyte, which is CD34-positive and CD31-negative, in the prostate stroma. It is possible to verify a telopode and the alternation between podomers and podoms (Fig. 5A–D). Another telocyte is observed, with a telopode reaching a prostate alveolus (Fig. 5E–H). In the prostate of elderly animals (24 months of age), CD34 immunostaining typical of a telocyte can be observed in the periepithelial region (Fig. 5I–L). Telocyte immunostaining is also observed close to the prostate alveoli in the interstitium (Fig. 5M–P). With regard to the double immunofluorescence assays for CD34 and IL-6, as well as for CD34 and TNF-α in histological sections of adult and elderly Mongolian gerbil males, it can be seen that, in the adult prostate, the labeling for CD34 colocalizes with that of IL-6 in blood vessels, but not in telocytes (Fig. 6A–D). Similarly, in the prostate of senile animals, the labeling for CD34 does not colocalize with IL-6 in telocytes. The colocalization only occurs in blood vessels (Fig. 6E–H). In the case of labeling for TNF-α, in the adult prostate, it colocalizes with CD34 in some blood vessels, but does not colocalize in the telocytes (Fig. 6I–L). This is also seen in the senile prostate (Fig. 6M–P).

**Figure 7:**
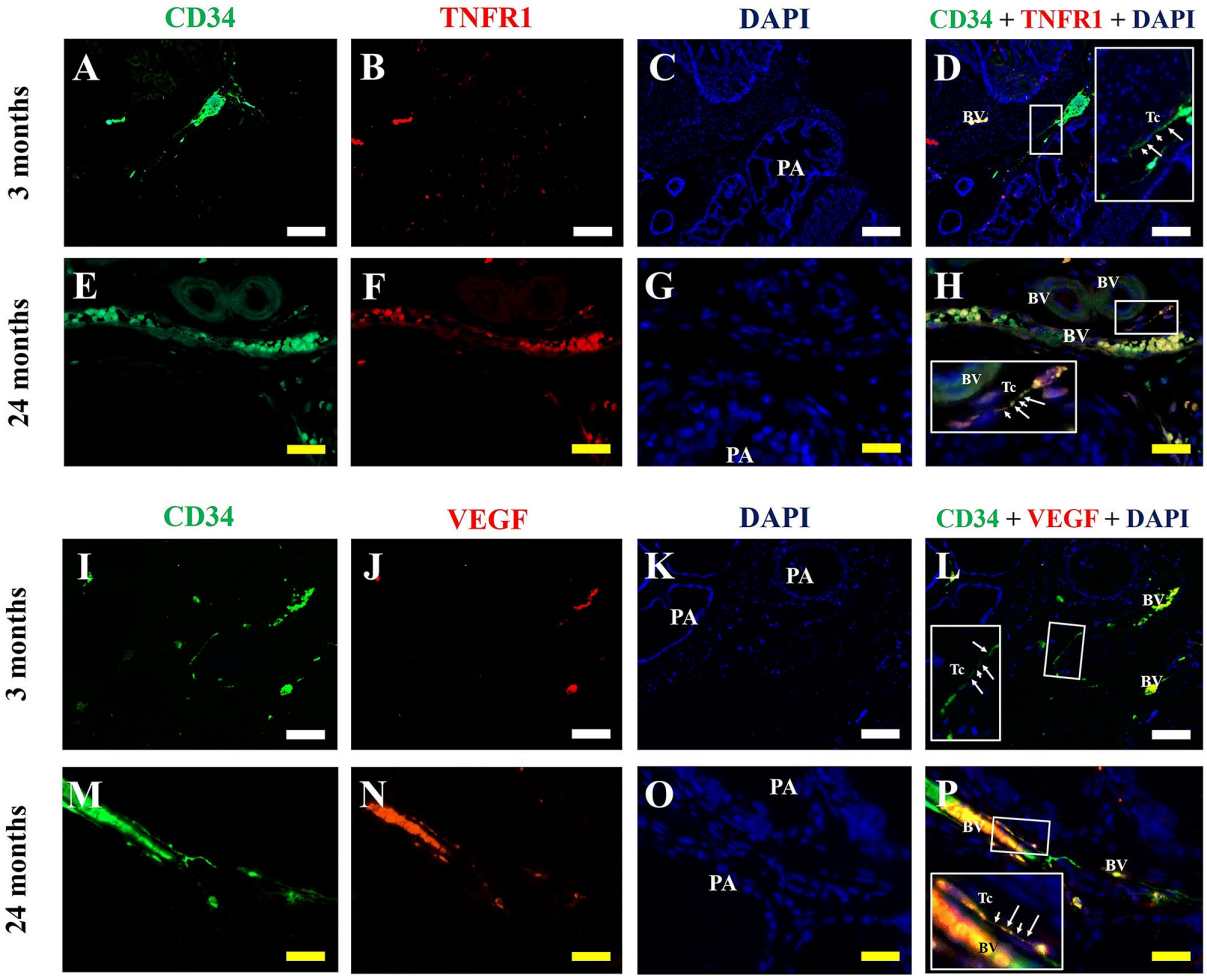
Double immunofluorescence assays for CD34 and TNFR1, as well as for CD34 and VEGF in prostate histological sections of the adult (3 months old) and senile (24 months old) male Mongolian gerbils. (**A**–**D**) In the adult prostate, TNFR1 does not colocalize in the telocytes. (**E**–**H**) In the senile prostate, it is possible to observe the TNFR1 immunolabeling in a telocyte. (**I**–**L**) The colocalization of VEGF immunolabeling with CD34 in the adult prostate can be seen in blood vessels, but not in telocytes. (**M**–**P**) In the senile prostate, it is possible to verify the colocalization between VEGF and CD34 in a telocyte. PA (Prostate alveoli), BV (Blood vessel), Tc (Telocyte), Insert (Detail of telocytes, in which the moniliform aspect of the telopodes can be observed), Arrow (Podoms), arrowhead (Podomers), White bar (100 µm), Yellow bar (50 µm).

**Figure 8:**
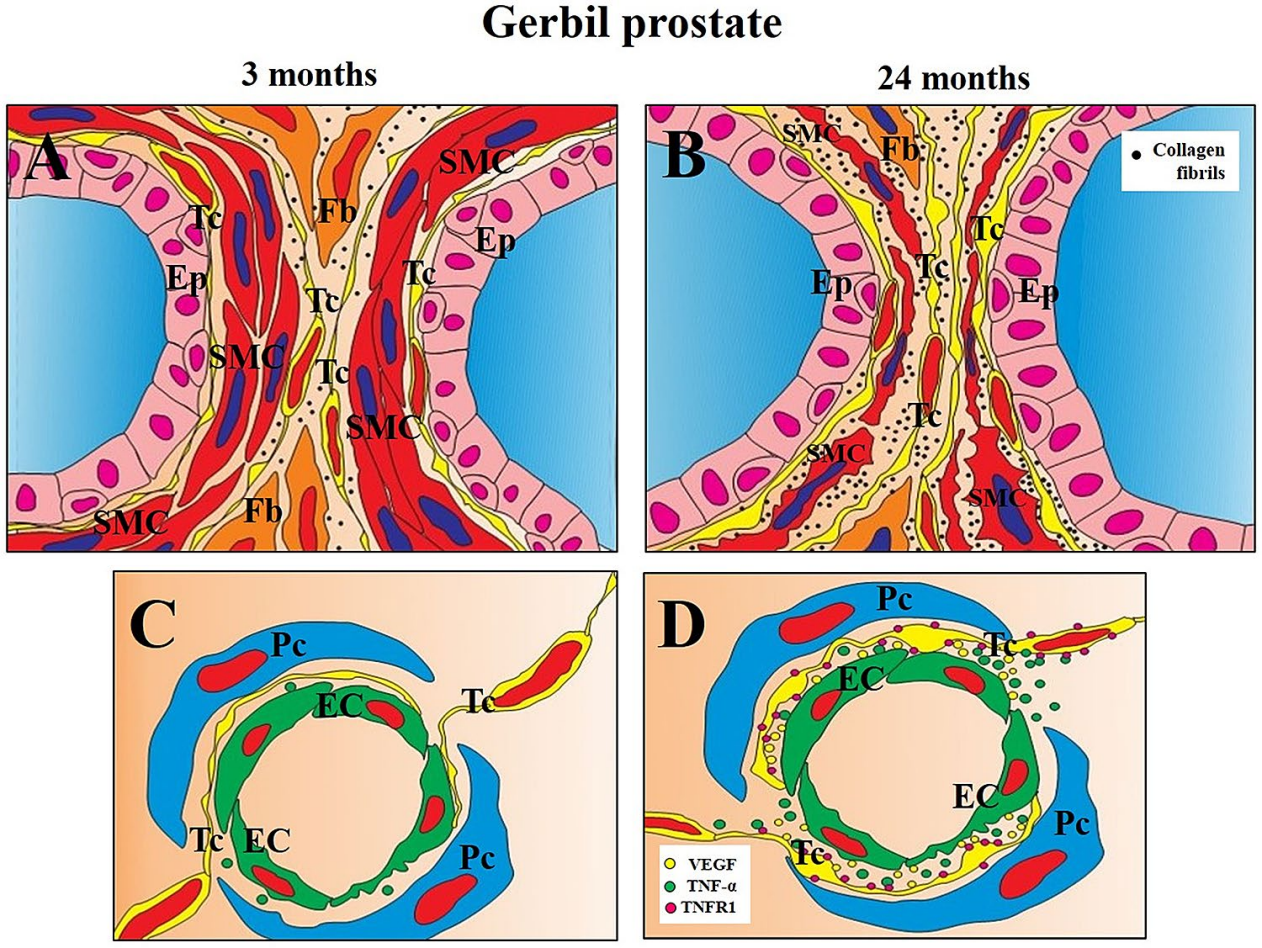
Schematic drawing depicting aspects of the aged prostate of male Mongolian gerbils and the telocytes ’relationships with other relevant tissue components. (**A**) The prostate of three-month-old animals (adults) has a compact layer of perialveolar smooth muscle; the telocytes occupy the periepithelial region, as well as the interstitium, surrounding the layer of perialveolar smooth muscle. (**B**) In the prostate of 24-month-old (senile) animals, the smooth muscle layers become thinner as the prostatic alveoli become more dilated. There is a great increase in the presence of extracellular matrix in the prostatic stroma, especially among smooth muscle cells. (**C**) In blood vessels of the adult prostate, telocytes establish cellular contacts with endothelial cells, and their cytoplasmic extensions, the telopodes, extend between the endothelial cells and the pericytes. (**D**) In the prostate of senile animals, telocytes secrete VEGF and are TNFR1-positive. Tc (Telocyte), Fb (Fibroblast), SMC (Smooth muscle cell), Ep (Prostate epithelium), Pc (Pericyte), EC (Endothelial cell).

Finally, with regard to double immunofluorescence assays for CD34 and TNFR1, as well as for CD34 and VEGF in prostate histological sections of adult and senile male Mongolian gerbils, in the adult prostate, TNFR-1 does not colocalize in telocytes (Fig. 7A–D). In the senile prostate, TNFR-1 labeling can be observed in telocytes (Fig. 7E–H). The colocalization of VEGF labeling with CD34 in the adult prostate can be seen in blood vessels, but not in telocytes (Fig. 7I–L). In the senile prostate, it is possible to verify the colocalization between VEGF and CD34 in telocytes (Fig. 7M–P). The relationship between ventral prostate modifications and telocytes during senescence is synthesized in Fig. 8.

## Discussion

Previous data evaluating prostate telocytes concerned animals that had undergone castration and these underwent major phenotypic changes, becoming atrophic^25^; in this sense, the decrease in the presence of testosterone after castration may be more impacting for telocytes than the aging process. In the present work, that avaluates telocytes during aging, these cells remain closely associated with the layers of perialveolar smooth muscle of the gerbil prostate throughout adult phase. But, unlike the human prostate, which has a fibromuscular stroma^46^, the gerbil prostate has well-defined layers of perialveolar smooth muscle in the adult phase, with little extracellular matrix. But, with senescence, the muscle layers become thinner and interspersed in the extracellular matrix, which indicates possible changes in their functionality. On the one hand, telocytes can assist in the production of elements of the extracellular matrix, together with fibroblasts, something hypothesized in other organs^8,47^. On the other hand, it may be possible that telocytes can contribute to maintaining the integrity of the smooth muscle layers, since our data indicate a network of telopodes that surrounds the prostatic alveoli. This network can generate mechanical forces capable of avoiding deformations in the alveoli, partially maintaining their functionalities, such as protecting the architecture of the perialveolar stroma. In this regard, there are recent data concerning the testicles, which indicate that telocytes form extensive networks occupying the peritubular and intertubular regions^48^, and changes in these networks, with the reduction in the number of telocytes in pathological conditions, contribute to the disorganization of the normal stromal architecture, so that the telocyte networks could be essential for the maintenance of tissue organization in the testicles^49^, something that our data also indicate for the senile prostate.

However, unlike perialveolar telocytes, which may possibly have a protective effect in the face of changes in the prostatic stroma, our data indicate that perivascular telocytes can act in the opposite direction, being related to two central processes in stromal changes seen in the senescent prostate. The first of these is angiogenesis and the other is the establishment of a pro-inflammatory microenvironment. We found that vascular telocytes in the prostate produce VEGF, which indicates the participation of these cells in angiogenesis. This contribution by the telocytes was also verified in the lung^3^ and in the mammary glands^50^. With regard to the establishment of a pro-inflammatory microenvironment, our data indicate that telocytes do not actively secrete pro-inflammatory cytokines, such as TNFα and IL-6, in the prostate. However, these cells produce TNFR1, which is the TNFα receptor. TNFα is secreted by the blood vessels of the senile prostate and telocytes are able to respond to it. This indicates that telocytes are sensitive and can possibly contribute to the pro-inflammatory environment that builds up in the prostate with aging. The pro-inflammatory role of telocytes has been previously verified^51^. Both angiogenic stimulation and TNFα sensitivity indicate that telocytes can contribute to the formation of so-called reactive stroma^52^.

In general, the present study indicates that telocytes may play an active role both in resisting and in promoting typical changes that occur with prostate aging. On the one hand, telocytes possibly help to maintain the integrity of the smooth muscle layers of prostatic alveoli, whose musculature undergoes a narrowing and an increase in the presence of extracellular matrix, losing its typical conformation with aging, and the telocyte networks, which were found to be closely related to this musculature, remain intact in order to contribute to the maintenance of the musculature and the stromal architecture. On the other hand, we found evidence that telocytes, especially vascular ones, may contribute to the establishment of a pro-angiogenic and inflammatory microenvironment that is verified in the gland with aging^29,37^. Regarding these changes, there is evidence that they can arise spontaneously in the senile prostate and that they can be maximized due to exposure to chemicals or specific diets^43,53^.

These aspects can contribute to the formation of a microenvironment that promotes most of the prostatic pathologies^54–56^, such as benign prostate hyperplasia or even prostate cancer. Telocytes are still largely ignored in prostate pathogenesis; although to a lesser extent than fibroblasts, telocytes are stromal cells that establish multiple interrelationships with other prostate cells, whether epithelial or stromal. Our data on the relationship between prostate aging and telocytes, in addition to data on the effects of castration on telocytes^25^, indicate that prostate telocytes are an important element in the understanding of prostate physiology, specifically regarding the maintenance of gland stroma organization and the pro-pathogenic variations that the prostate may undergo in the formation of so-called reactive stroma. In future research, an important topic to be addressed with regard to telocytes in the prostate and other organs of the urogenital system is the relationship between these and the stem cells in the stroma, something that has already been seen in other organs^57,58^, especially in the maintenance of the stem cell microenvironment.

In conclusion, our data show for the first time that, with aging, prostate telocytes form a network of telopodes around the prostatic alveoli, and can contribute to maintaining the integrity of the perialveolar muscle layers, as well as being able to promote angiogenesis through the synthesis of VEGF and to establish a pro-inflammatory microenvironment since they are sensitive to TNFα. Together, our data indicate that telocytes play an active role in the establishment of the typical changes that the prostate undergoes with aging. Our data also suggest that the study of these cells is promising for reaching an understanding of the onset of common prostate pathologies, such as benign prostate hyperplasia and prostate cancer, and in the search for new treatment targets for these diseases.

## Material and methods

### Animals

The animals were provided by São Paulo State University (UNESP, São José do Rio Preto). Adult male (3-months-old) and senile (24-months-old) Mongolian gerbils were housed in a temperature-controlled (25 °C) room on a 12 h light/dark cycle. All of the animals were housed in polyethylene cages, with ad libitum access to filtered water and rodent food. All experimental protocols were performed according to the ethical guidelines of São Paulo State University (UNESP). All the experimental protocols were approved by the ethics committee in the use of animal (CEUA) of the São Paulo State University (protocol 115/2015). Animals were killed by lethal injections containing a mixture of an anaesthetic, ketamine (100 mg/kg bw, Dopalen, Vetbrands, Brazil), and a muscle relaxant, xylazine (11 mg/kg bw, Rompun, Bayer, Brazil). The ventral prostate (VP) was excised and immediately immersed in fixative, as previously described^25^.

### Histological processing

After dissection, the prostate glands were collected and fixed in buffered 4% paraformaldehyde, washed in water, dehydrated in ethanol, clarified in xylol, and then embedded in Paraplast (Histosec, Merk). The organs were sectioned at 3–5 μm and histological sections were mounted; a portion of them was stained by the Hematoxylin–Eosin (H&E) histochemical technique for general morphological studies.

### Three-dimensional reconstructions

Three-dimensional (3D) reconstructions were performed for both adult and senile prostates in order to compare structural changes in the gland throughout the aging process. Histological sections were cut serially, 5 μm thick, and were then mounted in glass slides and stained with H&E. These were subsequently analysed and photographed using an Olympus BX60 light microscope (Olympus, Japan) coupled to a computer with DP-BSW V3.1 software (Olympus) AMS and Image Pro 6.1 software (Media Cybernetics) for Windows (Media Cybernetics, Silver Spring, MD). Later, the images were reconstructed using the software Reconstruct^59^.

### Transmission electron microscopy (TEM)

Ultrastructural analysis was performed using the protocol described by Corradi et al.^16^ Fragments of the adult and senile gerbil ventral prostates were minced into small pieces and fixed by immersion in 3% glutaraldehyde plus 0.25% tannic acid solution in Millonig’s buffer, pH 7.3, containing 0.54% glucose, for 24 h. After washing with the same buffer, samples were post‐fixed with 1% osmium tetroxide for 1 h, washed in buffer, dehydrated in a graded acetone series and embedded in Araldite resin. Ultrathin Sects. (50–75 nm) were prepared using a diamond knife and stained with 2% alcoholic uranyl acetate for 30 min. followed by 2% lead citrate in 1 M sodium hydroxide for 10 min. Samples were analyzed by electron microscopy using a Hitachi HT 7800 at 80 kV.

### Immunofluorescence of paraffin‐embedded tissue sections

The prostate samples were fixed in 4% paraformaldehyde (buffered in 0.1 M phosphate, pH 7.4) for 24 h. After fixing, the tissues were washed in water, dehydrated in a series of ethanol solutions, embedded in paraffin (Histosec; Merck, Darmstadt, Germany), then sectioned at 5 μm using a microtome (RM2155, Leica, Nussloch, Germany). In order to detect telocytes in the adult and senile prostate, tissue sections were subjected to double immunofluorescence assays for CD34 / CD31 (polyclonal mouse CD34 IgG, B ‐ 6, sc74499; rabbit polyclonal CD31 IgG, M ‐ 185, sc ‐ 28188; Santa Cruz Biotechnology, Dallas, TX, USA), as previously described^25^. To verify the disposition of perialveolar smooth muscle between the groups, tests were performed for α-SMA (α-SMA monoclonal mouse, IgG2a sc-130617, Santa Cruz Biotechnology, Dallas, TX, USA). In order to verify a possible inflammatory role for telocytes in the prostate, double immunofluorescence assays were performed for CD34 / TNFα (monoclonal mouse CD34, IgG, B-6, sc74499; goat polyclonal TNFα, IgG, N-19, sc-1350; Santa Cruz Biotechnology, Dallas, TX, USA), CD34/TNFR1 (monoclonal mouse CD34, IgG, B-6, sc74499; rabbit polyclonal TNFR1, IgG, H-271, sc-7895; Santa Cruz Biotechnology, Dallas, TX, USA ) and for CD34/IL-6 (monoclonal mouse CD34, IgG, B-6, sc74499; goat polyclonal, IgG, M-19, sc-1265; Santa Cruz Biotechnology, Dallas, TX, USA). Finally, in order to investigate the possible angiogenic role of telocytes, double immunofluorescence assays for CD34 / VEGF (monoclonal mouse CD34, IgG, B-6, sc74499; rabbit polyclonal, IgG, A-20, sc-152; Santa Cruz Biotechnology, Dallas, TX, USA) were carried out. These antibodies were incubated overnight at a 1:100 dilution. The following morning, sections were incubated with goat antimouse FITC-labeled (sc ‐ 2011; Santa Cruz Biotechnology), goat anti-rabbit Texas Red-labeled (sc ‐ 2780; Santa Cruz Biotechnology) or donkey anti-goat Texas Red-labeled (sc-2783; Santa Cruz Biotechnology) secondary antibodies, diluted 1: 200 in 1% bovine serum albumin (BSA) for 2 h at room temperature, then stained with DAPI (F36924; Life Technology, Grand Island, NY, USA). The histological sections were analyzed with a ZeissImager M2 fluorescence microscope (Zeiss, Oberkochen, Germany) coupled to AxioVision (Zeiss) software, as the routine^25^. The immunofluorescence assays for the negative controls were performed without the incubation with the antibodies and the other steps were the same aforementioned.
